# FishTEDB: a collective database of transposable elements identified in the complete genomes of fish

**DOI:** 10.1093/database/bax106

**Published:** 2018-01-16

**Authors:** Feng Shao, Jianrong Wang, Hongen Xu, Zuogang Peng

**Affiliations:** 1 Key Laboratory of Freshwater Fish Reproduction and Development (Ministry of Education), Southwest University School of Life Sciences, Chongqing 400715, China; 2 Department of Computational Mathematics, Science and Engineering, Michigan State University, MI 48824, USA; 3 Department of Genome Oriented Bioinformatics Wissenschaftszentrum Weihenstephan, TU Muenchen Maximus-von-Imhof-Forum 3, Freising 85354, Germany

## Abstract

Transposable elements (TEs) are important for host gene regulation and genome evolution. Consensus sequences of TEs can assist investigators in accelerating studies on TE origins, amplification, functions and evolution, as well as comparative analyses and prediction of TEs in different species. In evolution, physiology, ecology and heredity research, fish are important models. However, to date, no comprehensive resource for TE consensus sequences exists for fish. Here, we collected genome-wide data and developed a novel database, FishTEDB, including 27 bony fishes, 1 cartilaginous fish, 1 lamprey and 1 lancelet. *De novo*, structure-based and homology-based approaches were combined to detect TEs. The database is open-source and user-friendly, and users can browse, search and download all data. FishTEDB also provides GetORF, BLAST and HMMER tools to analyze sequences.

**Database URL**: http://www.fishtedb.org/

## Introduction

Transposable elements (TEs) are discrete DNA segments that can insert into new chromosomal locations by one of two mechanisms (1). TEs are typically divided into Class I (‘copy and paste’ style, retrotransposons) and Class II (‘cut and paste’ style, transposons) based on whether the intermediate they use to move is RNA or DNA (2). On the basis of sequence similarities and structural relationships, these classes can be further subdivided into orders and superfamilies. Retrotransposons are commonly grouped into five distinct orders: long terminal repeat (LTR), *Dictyostelium intermediate* repeat sequence (DIRS), *Penelope*-like element (PLE), long interspersed nuclear element (LINE) and short interspersed nuclear element (SINE). DNA transposons consist of four main orders: terminal inverted repeat (TIR), Helitron, Crypton and Maverick (3). TEs are commonly considered molecular parasites owing to their removable and reproducible characteristics. However, studies of TEs in the past several decades have shown that transposons can affect gene regulation, function and coding ability (4–6). Transposons also play important roles in new gene creation, chromosome rearrangement and genome evolution (7–11). Recently, the regulatory activities of TEs in both plants and animals have become a focus of research. For example, in the peppered moth, TEs enhance *cortex* gene expression levels, which underlies the adaptive coloration that occurred during the industrial revolution (12). In oil palms, sporadic demethylation of a *Karma* TE within an intron of the *MANTLED* gene caused the mantled fruit phenotype (13).

Fish are the largest and oldest group of vertebrates. Thus far, 33 700 species have been recorded in Fishbase (http://www.fishbase.org/, version 10/2017), and this number is constantly increasing. Fish play a crucial role in modern biology. For example, zebrafish are not only model organisms for developmental biology but also a major disease research model (14, 15). Lungfish and coelacanth, which have been described as ‘living fossils’, provide a unique opportunity to understand the mechanisms that enabled the successful adaptation of vertebrates to land (16, 17). The content, diversity and distribution of TEs in fish genomes have been studied (18–21); however, the functions and evolutionary significance of transposons in fish genomes are largely unknown. A comprehensive database of fish TEs is needed to facilitate studies of TE functions and evolution in fish genomes.

In this study, we identified 33 260 consensus sequences of TEs classified into ∼50 superfamilies from 28 fish species, 1 lamprey and 1 lancelet, using *de novo*, structure-based and homology-based approaches. We integrated all data into a centralized database, FishTEDB, which allows users to browse, search and download all data. In addition, the GetORF, BLAST and HMMER web-based tools were provided to facilitate analyses of genomic sequences. FishTEDB can be used not only to study the origin, amplification mechanism and evolutionary dynamics of TEs in fish, but also for comparative analyses among vertebrates to elucidate the roles of TEs on genes and genomes.

## Materials and methods

### Data collection

All fish, lancelet and lamprey genomes used in this study were downloaded from public databases (Table 1). The Repbase Update collection (update 20150807) was retrieved from http://www.girinst.org/repbase/index.html (22). The Swiss-Prot data were downloaded from http://www.uniprot.org/downloads (23).

**Table 1.** Species in FishTEDB and their genome websites

Species	Download links
*Anguilla anguilla*	https://www.ncbi.nlm.nih.gov/assembly/GCA_000695075.1
*Anguilla japonica*	https://www.ncbi.nlm.nih.gov/assembly/GCA_000470695.1
*Astyanax mexicanus*	ftp://ftp.ensembl.org/pub/release-84/fasta/astyanax_mexicanus/dna/
*Branchiostoma floridae*	http://mosas.sysu.edu.cn/genome/download_data.php
*Callorhinchus milii*	http://esharkgenome.imcb.a-star.edu.sg/
*Ctenopharyngodon idellus*	http://www.ncgr.ac.cn/grasscarp/
*Cynoglossus semilaevis*	https://www.ncbi.nlm.nih.gov/assembly/GCA_000523025.1
*Dicentrarchus labrax*	https://www.ncbi.nlm.nih.gov/assembly/GCA_000689215.1
*Electrophorus electricus*	http://efishgenomics.zoology.msu.edu/? q=node/1
*Gadus morhua*	ftp://ftp.ensembl.org/pub/release-84/fasta/gadus_morhua/dna/
*Gasterosteus aculeatus*	ftp://ftp.ensembl.org/pub/release-84/fasta/gasterosteus_aculeatus/dna/
*Larimichthys crocea*	https://www.ncbi.nlm.nih.gov/assembly/GCA_000972845.1
*Lates calcarifer*	https://www.ncbi.nlm.nih.gov/assembly/GCA_001010145.1
*Latimeria chalumnae*	ftp://ftp.ensembl.org/pub/release-84/fasta/latimeria_chalumnae/dna/
*Lepisosteus oculatus*	ftp://ftp.ensembl.org/pub/release-84/fasta/lepisosteus_oculatus/dna/
*Neolamprologus brichardi*	https://www.ncbi.nlm.nih.gov/assembly/GCA_000239395.1
*Nothobranchius furzeri*	http://africanturquoisekillifishbrowser.org/downloads.html
*Notothenia coriiceps*	https://www.ncbi.nlm.nih.gov/assembly/GCA_000735185.1
*Oreochromis niloticus*	ftp://ftp.ensembl.org/pub/release-84/fasta/oreochromis_niloticus/dna/
*Oryzias latipes*	ftp://ftp.ensembl.org/pub/release-84/fasta/oryzias_latipes/dna/
*Periophthalmus magnuspinnatus*	https://www.ncbi.nlm.nih.gov/assembly/GCA_000787105.1
*Petromyzon marinus*	ftp://ftp.ensembl.org/pub/release-84/fasta/petromyzon_marinus/dna/
*Poecilia formosa*	ftp://ftp.ensembl.org/pub/release-84/fasta/poecilia_formosa/dna/
*Scleropages formosus*	https://www.ncbi.nlm.nih.gov/assembly/GCA_001005745.2
*Sinocyclocheilus graham*	https://www.ncbi.nlm.nih.gov/assembly/GCA_001515645.1
*Takifugu flavidus*	https://www.ncbi.nlm.nih.gov/assembly/GCA_000400755.1
*Takifugu rubripes*	ftp://ftp.ensembl.org/pub/release-84/fasta/takifugu_rubripes/dna/
*Tetraodon nigroviridis*	ftp://ftp.ensembl.org/pub/release-84/fasta/tetraodon_nigroviridis/dna/
*Thunnus orientalis*	https://www.ncbi.nlm.nih.gov/assembly/GCA_000418415.1
*Xiphophorus maculates*	ftp://ftp.ensembl.org/pub/release-84/fasta/xiphophorus_maculatus/dna/

### Collection and identification of TEs in fish genomes

**Figure 1.**
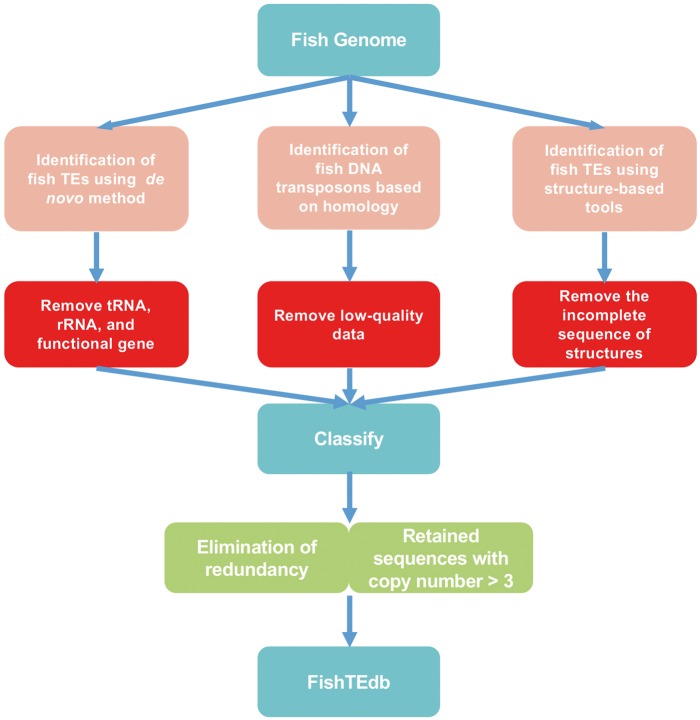
Flowchart of the TE analysis pipeline.

TE libraries of fish were generated using *de novo*, homology-based and structure-based methods (Figure 1). *De novo* identification of TEs was performed using RepeatModeler (http://www.repeatmasker.org/RepeatModeler/, version 1.0.7), which assists in automating the runs of RECON (24) and RepeatScout (25) to analyze fish genomic databases, and the output of this software was used to build, refine and classify consensus models of putative interspersed repeats. Repeats identified by RepeatModeler were filtered for tandem repeat coverage of >25%, using Tandem Repeats Finder (http://tandem.bu.edu/trf/trf.unix.help.html, version 4.07b) with the default parameters. The preserved sequences were used as queries for BlastX (identity > 30%, e-value < 1e-5 and percent query coverage > 50%) to search against Swiss-Prot data to filter protein-coding genes. We constructed a library of ncRNAs using tRNAscan-SE (version 1.3.1) (26) and Rfam (27) to filter tRNA and rRNA by Blastn (identity > 90%, BLAST e-value < 1e-5 and percent query coverage > 90%).

For the LTR and non-LTR retroelements, given their easier-to-detect structural peculiarities (3), a structure-based approach was used. For LTR retrotransposons, LTR_STRUC (28) and MGEScan-LTR (http://darwin.informatics.indiana.edu/cgi-bin/evolution/daphnia_ltr.pl) were used to search the assembly of fish genomes with default parameters. For the MGEScan-LTR, intact LTR retroelements were identified using multiple empirical rules: similarity of a pair of LTRs at both ends, structure with internal regions (IRs), di (tri)-nucleotides at flanking ends and target site duplications (TSDs). We only retained the results that had these four structures. This framework was applied to identify a large number of novel elements, which were later analyzed to estimate the evolutionary history and relationships of LTR retrotransposons. Non-LTR retrotransposons were identified by the pHMM-based MGEScan-non-LTR (29) program with default parameters.

Given that Class II TEs lack easy-to-detect structural features, a homology-based method using TESeeker was employed to predict them. TESeeker is an automated homology-based approach for identifying TEs that is BLAST-based, but also makes use of the CAP3 assembly program and the ClustalW2 multiple sequence alignment tool, as well as numerous BioPerl scripts (30). In total, 257 transposase protein sequences from fish DNA transposons were extracted from RepBase and NCBI. These sequences were used as the library in TESeeker. Finally, we only retained the sequences with the highest quality in the consensus_contigs.fas file.

### TE classification and redundancy elimination in fish genomes

When identifying TEs in fish genomes, some software (TESeeker, RepeatModeler, MGEScan-LTR) can classify TEs in superfamilies, but the classification of some sequences remains unknown. REPCLASS (version 1.0, https://github.com/feschottelab/REPCLASS) and TEclass (31) were used to classify these TEs. REPCLASS is the first software used for classification of TEs. It uses an automated high-throughput workflow model, leveraging various programs to identify and classify TEs in new genomes. REPCLASS can classify consensus sequences into superfamilies. TEclass uses a machine learning support vector machine (SVM) for classification based on oligomer frequencies to classify unknown TEs into DNA transposons, LTRs, LINEs and SINEs (31). Hence, for the consensus sequences that cannot be classified into a superfamily by REPCLASS, we used TEclass (http://www.compgen.unimuenster.de/tools/teclass/generate/index.pl?lang=en) to classify them into orders.

In the step of TE prediction, we combined all of the results directly in a ‘union’ set of different types of evidence; therefore, the results contained redundant TEs that were predicted based on different methods. We reduced the presence of redundant sequences by CD-HIT (32) with parameters cd-hit-est -c 0.90 and –n 8. Some transposons may insert in or next to other retrotransposons (especially in LTR), forming highly TE-rich regions (Nested TEs) (33–35). For example, some DNA transposons may insert into LTR. Normally, if all the results are put together for filtering, DNA transposons are filtered out because they are shorter than LTR. Thus, to prevent interference by nested TEs, we removed redundancies from the superfamily units one by one. We aligned the sequences that could not be classified into superfamily level (‘Unknown’ elements) to corresponding genomes by BLAST (identity > 85% and coverage > 50%), and only retained sequences with copy number > 3.

### Implementation and web interface

**Figure 2.**
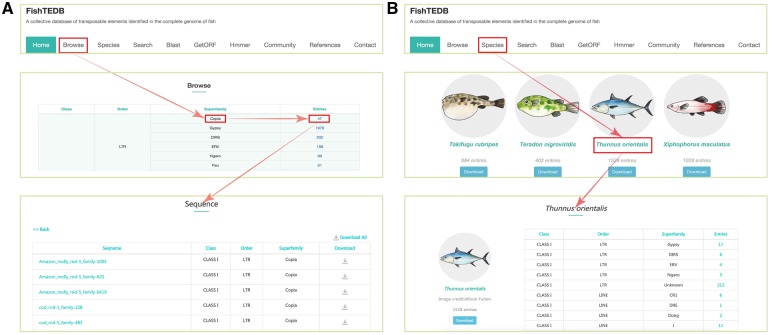
User interface introduction. (**A**) Browsing data shown in a superfamily-centric way; (**B**) Browsing data shown in a species-centric way.

To make this vast amount of TE data available, a user-friendly web-based database, FishTEDB, was constructed. FishTEDB enables users to browse, search, download and analyze TEs (Figure 2). FishTEDB was constructed using Yii 2.0 (a high-performance PHP MVC framework for developing Web 2.0 applications). We used the Linux (CentOS 6.7) system as the server, Nginx 1.10 (a high-performance HTTP server and reverse proxy server) as the web server, Mysql 5.7 as the storage engine and PHP 7.0 for web development. Bootstrap 3.3, JavaScript, Jquery and HTML5 were also used for the web page.

### Browser

**Figure 3.**
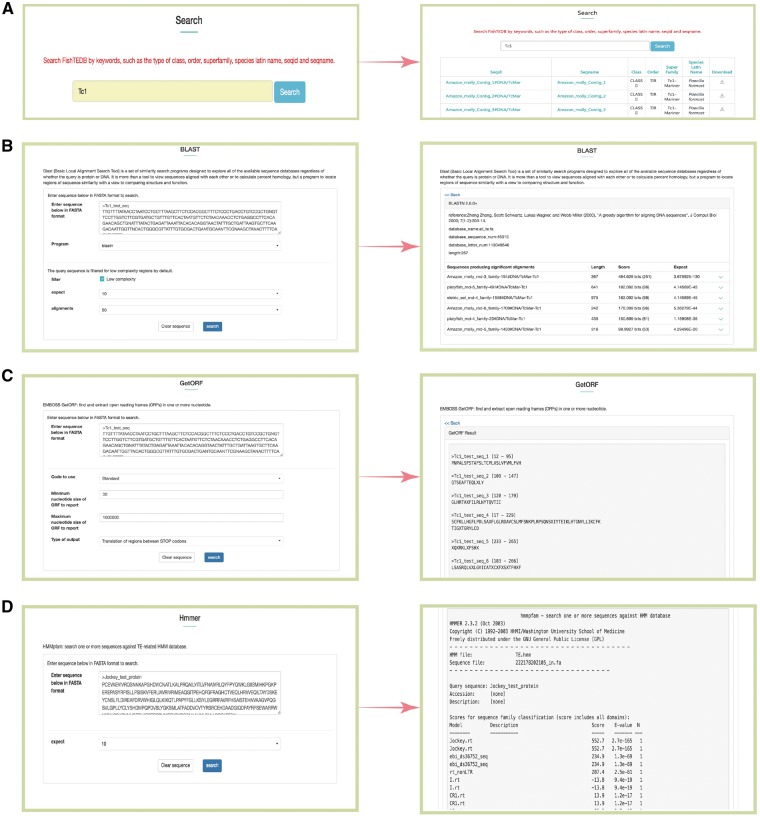
Snapshots of different functional sections provided in FishTEDB. (**A**) Screenshot of a keyword search results; (**B**) BLAST interface and a sample of BLASTn results; (**C**) GetORF interface and output results; (**D**) HMMER interface of a test protein sequence in FishTEDB.

**Figure 4.**
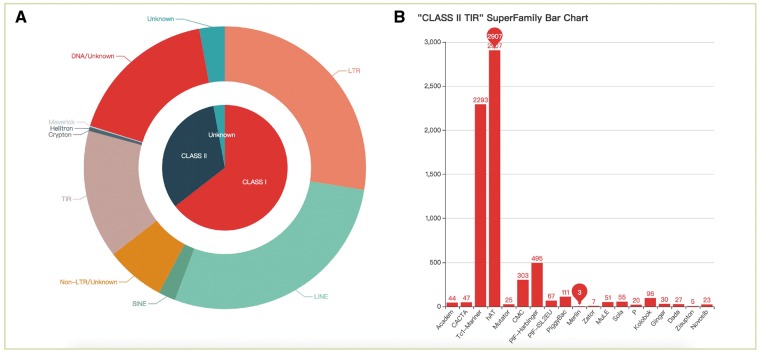
The statistics of consensus sequences. (**A**) Pie chart of different classes and orders; (**B**) Histogram of different superfamilies in TIR.

All TEs were displayed in the browsing interface in species- and superfamily-centric manners. Users can browse by superfamily by clicking the corresponding number. Detailed information for each superfamily can be retrieved using the hyperlinks provided (Figure 2A). In the species-centric interface, all TEs were assigned to corresponding species. In both interfaces, the same method was used to browse TE data (Figure 2B). Users can also use a keyword (TE class, TE order, TE superfamily, species name) to locate entries in the search section that used approximate string matching to implement (Figure 3A). All data can be downloaded. In addition, we calculated the number of different superfamily sequences and displayed it with a pie chart and histogram (Figure 4).

### Tools

Three general sequence analysis tools, that is, BLAST (36), GetORF (37) and HMMER (38), were further configured into our database.

- BLAST was used for the homology search, and users can align interest query sequences against FishTEDB to make an incipient judgment (whether the query sequence is a TE and which type it belongs to). BLAST will act as an efficient helper for researchers to detect whether TEs exist in sequences upstream and downstream sequences of genes of interest.
- Users can identify the potential open reading frame (ORF) in query sequences using the GetORF tool. Given that some TEs show differences (especially interspecies) even though they belong to the same superfamily, the results of the BLAST alignment may be deficient. GetORF can predict amino acid sequences (transposase, integrase, reverse transcriptase), and can be combined with BLAST and HMMER for TE identification and classification in species distantly related to fish at the nucleotide level.
- HMMER was used for the identification of transposase, endonuclease and reverse transcriptase domains of transposons. All profile-HMM (profile hidden Markov model) databases were collected from previous study (29) and Pfam (39).

Examples of BLASTN, GetORF and HMMER results are shown in Figure 3B–D, respectively.

## Results and discussion

In the seminal work of Barbara McClintock, TEs were proposed as the ‘controlling elements’ of maize (40). Since then, many researchers have paid close attention to the functions of TEs; however, to what extent the pervasive colonization of genomes by TEs has affected the evolution of eukaryotic gene regulation remains a matter of speculation and controversy (41). The evolution of fish began ∼530 million years ago during the Cambrian explosion (42). It was during this time that the early vertebrates developed the skull and the vertebral column, leading to the first vertebrates (43). Thus, supposing a TE mechanism, investigation of the roles of TEs in the genome evolution and the impact on host genes in fish may offer insights for other vertebrates. In this study, we constructed an effective combined pipeline, suitable not only for fish but also for other vertebrates. FishTEDB provides a good basis for TE functional studies and has an auxiliary role. First, FishTEDB can enrich the transposon data of vertebrates and promote transposon research. In particular, it would provide a homologous database for the identification and classification of TEs. Second, researchers can combine tools in FishTEDB with their own sequences to achieve rapid positioning of potential TEs.

We identified 33 260 TEs from 30 species: 28 fishes, 1 lamprey and 1 lancelet. Most TEs were classified into known superfamilies (Table 2). In addition, the results suggest that TEs are diverse in fish genomes. In particular, the *Gypsy*, *L1*, *L2*, *R2*, *RTE*, *Rex*, *Tc1-Mariner* and *hAT* superfamilies showed higher diversity than other superfamilies. Nevertheless, fishes and lancelet presented a lower diversity of SINEs.

**Table 2.** Summary of identified transposable elements families (/consensi) in FishTEDB

Class	Order	Superfamily	Quantity		
			Fish	Lamprey	Lancelet
CLASS I	LTR	*Copia*	45	1	1
		*Gypsy*	1787	160	29
		*DIRS*	199	*N*	3
		*ERV*	187	1	*N*
		*Ngaro*	91	6	2
		*Pao*	57	4	*N*
		Unknown LTR	3378	214	117
	LINE	*CR1*	611	98	171
		*CRE*	*N*	1	*N*
		*DRE*	3	*N*	*N*
		*Dong*	99	*N*	*N*
		*I*	210	5	8
		*Jockey*	29	28	5
		*L1*	2325	31	57
		*L2*	2794	75	72
		*Penelope*	171	69	15
		*Proto2*	18	*N*	7
		*R1*	5	1	1
		*R2*	626	10	21
		*RTE*	963	384	193
		*Rex*	954	48	39
		*Tad1*	7	1	1
		Unknown LINE	1379	21	86
	SINE	*5S*	41	4	*N*
		*7SL*	1	*N*	*N*
		*ID*	10	*N*	*N*
		*MIR*	75	*N*	13
		*U*	3	1	*N*
		*tRNA*	198	44	11
		Unknown SINE	347	5	19
		Unknown non-LTR	1879	43	98
CLASS II	TIR	*Academ*	20	3	21
		*CACTA*	45	*N*	2
		*Tc1-Mariner*	2224	58	11
		*hAT*	2804	52	51
		*Mutator*	15	*N*	10
		*CMC*	277	6	20
		*PIF-Harbinger*	438	1	56
		*PIF-ISL2EU*	63	1	3
		*PiggyBac*	94	*N*	17
		*Merlin*	3	*N*	*N*
		*Zator*	1	4	2
		*MuLE*	42	1	8
		*Sola*	45	2	8
		*P*	20	*N*	*N*
		*Kolobok*	96	*N*	*N*
		*Ginger*	19	*N*	11
		*Dada*	23	*N*	4
		*Zisupton*	5	*N*	*N*
		*Novosib*	21	*N*	2
	Crypton	*Crypton*	27	*N*	*N*
	Helitron	*Helitron*	162	22	3
	Maverick	*Maverick*	59	*N*	*N*
		Unknown DNA	4671	57	190
Unknown	Unknown	Unknown	678	14	52
Total			30344	1476	1440

Note. Numbers represent the number of consensus sequences and *N* indicates undetected.

It should be noted that we only classified ∼60% of consensus sequences in superfamilies. There are still many TEs that cannot be classified into known superfamilies. The karyotypes and genome sizes in fish are more diverse and complex than those of other vertebrates, and an extra level of complexity was observed due to whole genome duplication (WGD) and a rediploidization event that teleost fish have underwent during evolution (44). Therefore, we speculate that there are many fish-specific transposons, such as *Zisupton* (45). TE research is difficult without using a dedicated database. The transposon information of zebrafish in RepBase is probably the most comprehensive thus far, but that is still not sufficient to assist the classification of fish TEs. Nevertheless, these TEs may have potential effects on regulating host gene function and expression. In future studies, we will focus on the identification of novel superfamilies to further enrich TE data resources.

## References

[1] Mandal P.K., Kazazian H.H. (2008) SnapShot: vertebrate transposons. Cell, 135, 192–192.e191. 18854165 10.1016/j.cell.2008.09.028

[2] Finnegan D.J. (1989) Eukaryotic transposable elements and genome evolution. Trends Genet., 5, 103–107. 2543105 10.1016/0168-9525(89)90039-5

[3] Wicker T., Sabot F., Hua-Van A. (2007) A unified classification system for eukaryotic transposable elements. Nat. Rev. Genet., 8, 973–982. 17984973 10.1038/nrg2165

[4] Biemont C., Vieira C. (2006) Genetics: junk DNA as an evolutionary force. Nature, 443, 521–524. 10.1038/443521a 17024082

[5] Capy P. (1997) Evolution and Impact of Transposable Elements. Kluwer Academic Publishers, Dordrecht, The Netherlands.

[6] Finnegan D.J. (1992) Transposable elements. Curr. Opin. Genet. Dev., 2, 861–867. 1335807 10.1016/s0959-437x(05)80108-x

[7] Bennetzen J.L. (2000) Transposable element contributions to plant gene and genome evolution. Plant Mol. Biol., 42, 251–269. 10688140

[8] Bennetzen J.L. (2005) Transposable elements, gene creation and genome rearrangement in flowering plants. Curr. Opin. Genet. Dev., 15, 621–627. 16219458 10.1016/j.gde.2005.09.010

[9] Bucher E., Reinders J., Mirouze M. (2012) Epigenetic control of transposon transcription and mobility in *Arabidopsis*. Cur. Opin. Plant Biol., 15, 503–510. 10.1016/j.pbi.2012.08.006 22940592

[10] Feschotte C. (2008) Transposable elements and the evolution of regulatory networks. Nat. Rev. Genet., 9, 397–405. 18368054 10.1038/nrg2337 PMC2596197

[11] Long M., Betran E., Thornton K. (2003) The origin of new genes: glimpses from the young and old. Nat. Rev. Genet., 4, 865–875. 14634634 10.1038/nrg1204

[12] Van't Hof A.E., Campagne P., Rigden D.J. (2016) The industrial melanism mutation in British peppered moths is a transposable element. Nature, 534, 102–105. 10.1038/nature17951 27251284

[13] Ong-Abdullah M., Ordway J.M., Jiang N. (2015) Loss of Karma transposon methylation underlies the mantled somaclonal variant of oil palm. Nature, 525, 533–537. 10.1038/nature15365 26352475 PMC4857894

[14] Howe K., Clark M.D., Torroja C.F. (2013) The zebrafish reference genome sequence and its relationship to the human genome. Nature, 496, 498–503. 10.1038/nature12111 23594743 PMC3703927

[15] Kettleborough R.N., Busch-Nentwich E.M., Harvey S.A. (2013) A systematic genome-wide analysis of zebrafish protein-coding gene function. Nature, 496, 494–497. 23594742 10.1038/nature11992 PMC3743023

[16] Amemiya C.T., Alfoldi J., Lee A.P. (2013) The African coelacanth genome provides insights into tetrapod evolution. Nature, 496, 311–316. 10.1038/nature12027 23598338 PMC3633110

[17] Biscotti M.A., Gerdol M., Canapa A. (2016) The lungfish transcriptome: a glimpse into molecular evolution events at the transition from water to land. Sci. Rep., 6, 21571. 26908371 10.1038/srep21571 PMC4764851

[18] Capriglione T., Odierna G., Caputo V. (2002) Characterization of a *Tc1*-like transposon in the Antarctic ice-fish, *Chionodraco hamatus*. Gene, 295, 193–198. 10.1016/S0378-1119(02)00729-1 12354653

[19] Chalopin D., Naville M., Plard F. (2015) Comparative analysis of transposable elements highlights mobilome diversity and evolution in vertebrates. Genome Biol. Evol., 7, 567–580. 25577199 10.1093/gbe/evv005 PMC4350176

[20] Gao B., Shen D., Xue S. (2016) The contribution of transposable elements to size variations between four teleost genomes. Mob. DNA, 7, 4. 26862351 10.1186/s13100-016-0059-7 PMC4746887

[21] Schemberger M.O., Nogaroto V., Almeida M.C. (2016) Sequence analyses and chromosomal distribution of the *Tc1/Mariner* element in Parodontidae fish (Teleostei: Characiformes). Gene, 593, 308–314. 10.1016/j.gene.2016.08.034 27562083

[22] Jurka J., Kapitonov V.V., Pavlicek A. (2005) Repbase Update, a database of eukaryotic repetitive elements. Cytogenet. Genome Res., 110, 462–467. 16093699 10.1159/000084979

[23] Bairoch A., Apweiler R. (1999) The SWISS-PROT protein sequence data bank and its supplement TrEMBL in 1999. Nucleic Acids Res., 27, 49–54. 9847139 10.1093/nar/27.1.49 PMC148094

[24] Bao Z., Eddy S.R. (2002) Automated *de novo* identification of repeat sequence families in sequenced genomes. Genome Res., 12, 1269–1276. 12176934 10.1101/gr.88502 PMC186642

[25] Price A.L., Jones N.C., Pevzner P.A. (2005) *De novo* identification of repeat families in large genomes. Bioinformatics, 21(Suppl. 1), i351–i358. 15961478 10.1093/bioinformatics/bti1018

[26] Lowe T.M., Eddy S.R. (1997) tRNAscan-SE: a program for improved detection of transfer RNA genes in genomic sequence. Nucleic Acids Res., 25, 955–964. 9023104 10.1093/nar/25.5.955 PMC146525

[27] Nawrocki E.P., Burge S.W., Bateman A. (2015) Rfam 12.0: updates to the RNA families database. Nucleic Acids Res., 43, D130–D137. 25392425 10.1093/nar/gku1063 PMC4383904

[28] McCarthy E.M., McDonald J.F. (2003) LTR_STRUC: a novel search and identification program for LTR retrotransposons. Bioinformatics, 19, 362–367. 10.1093/bioinformatics/btf878 12584121

[29] Rho M., Tang H. (2009) MGEScan-non-LTR: computational identification and classification of autonomous non-LTR retrotransposons in eukaryotic genomes. Nucleic Acids Res., 37, e143. 19762481 10.1093/nar/gkp752 PMC2790886

[30] Kennedy R.C., Unger M.F., Christley S. (2011) An automated homology-based approach for identifying transposable elements. BMC Bioinformatics, 12, 130. 10.1186/1471-2105-12-130 21535899 PMC3107183

[31] Abrusan G., Grundmann N., DeMester L. (2009) TEclass – a tool for automated classification of unknown eukaryotic transposable elements. Bioinformatics, 25, 1329–1330. 10.1093/bioinformatics/btp084 19349283

[32] Huang Y., Niu B., Gao Y. (2010) CD-HIT Suite: a web server for clustering and comparing biological sequences. Bioinformatics, 26, 680–682. 10.1093/bioinformatics/btq003 20053844 PMC2828112

[33] Gao C., Xiao M., Ren X. (2012) Characterization and functional annotation of nested transposable elements in eukaryotic genomes. Genomics, 100, 222–230. 10.1016/j.ygeno.2012.07.004 22800764

[34] SanMiguel P., Tikhonov A., Jin Y.K. (1996) Nested retrotransposons in the intergenic regions of the maize genome. Science, 274, 765–768. 10.1126/science.274.5288.765 8864112

[35] Wei L., Xiao M., An Z. (2013) New insights into nested long terminal repeat retrotransposons in *Brassica* species. Mol. Plant, 6, 470–482. 22930733 10.1093/mp/sss081

[36] Altschul S.F., Madden T.L., Schaffer A.A. (1997) Gapped BLAST and PSI-BLAST: a new generation of protein database search programs. Nucleic Acids Res., 25, 3389–3402. 9254694 10.1093/nar/25.17.3389 PMC146917

[37] Rice P., Longden I., Bleasby A. (2000) EMBOSS: the European Molecular Biology Open Software Suite. Trends Genet., 16, 276–277. 10827456 10.1016/s0168-9525(00)02024-2

[38] Finn R.D., Clements J., Eddy S.R. (2011) HMMER web server: interactive sequence similarity searching. Nucleic Acids Res., 39, W29–W37. 21593126 10.1093/nar/gkr367 PMC3125773

[39] Finn R.D., Coggill P., Eberhardt R.Y. (2016) The Pfam protein families database: towards a more sustainable future. Nucleic Acids Res., 44, 279–285. 10.1093/nar/gkv1344 PMC4702930 26673716

[40] McClintock B. (1956) Controlling elements and the gene. Harb. Symp. Quant. Biol., 21, 197–216. 10.1101/sqb.1956.021.01.017 13433592

[41] Chuong E.B., Elde N.C., Feschotte C. (2017) Regulatory activities of transposable elements: from conflicts to benefits. Nat. Rev. Genet., 18, 71–86. 27867194 10.1038/nrg.2016.139 PMC5498291

[42] Clarke T. (2002). Oldest fossil footprints on land. *Nature*.

[43] Shu D.G., Morris S.C., Han J. (2003) Head and backbone of the early Cambrian vertebrate *Haikouichthys*. Nature, 421, 526–529. 10.1038/nature01264 12556891

[44] Volff J. (2005) Genome evolution and biodiversity in teleost fish. Heredity, 94, 280–294. 10.1038/sj.hdy.6800635 15674378

[45] Bohne A., Zhou Q., Darras A. (2012) *Zisupton*—a novel superfamily of DNA transposable elements recently active in fish. Mol. Biol. Evol., 29, 631–645. 21873630 10.1093/molbev/msr208

